# Reconstruction of the pulmonary artery by a novel biodegradable conduit engineered with perinatal stem cell-derived vascular smooth muscle cells enables physiological vascular growth in a large animal model of congenital heart disease

**DOI:** 10.1016/j.biomaterials.2019.119284

**Published:** 2019-10

**Authors:** Mohamed T. Ghorbel, Huidong Jia, Megan M. Swim, Dominga Iacobazzi, Ambra Albertario, Carlo Zebele, Delphine Holopherne-Doran, Anthony Hollander, Paolo Madeddu, Massimo Caputo

**Affiliations:** a Bristol Heart Institute, School of Clinical Sciences, University of Bristol, Bristol, UK; b Institute of Integrative Biology, University of Liverpool, Liverpool, UK

**Keywords:** Congenital heart disease, Stem cells, Tissue-engineering, Grafts

## Abstract

Lack of growth potential of available grafts represents a bottleneck in the correction of congenital heart defects. Here we used a swine small intestinal submucosa (SIS) graft functionalized with mesenchymal stem cell (MSC)-derived vascular smooth muscle cells (VSMCs), for replacement of the pulmonary artery in piglets.

MSCs were expanded from human umbilical cord blood or new-born swine peripheral blood, seeded onto decellularized SIS grafts and conditioned in a bioreactor to differentiate into VSMCs. Results indicate the equivalence of generating grafts engineered with human or swine MSC-derived VSMCs. Next, we conducted a randomized, controlled study in piglets (12–15 kg), which had the left pulmonary artery reconstructed with swine VSMC-engineered or acellular conduit grafts. Piglets recovered well from surgery, with no casualty and similar growth rate in either group. After 6 months, grafted arteries had larger circumference in the cellular group (28.3 ± 2.3 vs 18.3 ± 2.1 mm, P < 0.001), but without evidence of aneurism formation. Immunohistochemistry showed engineered grafts were composed of homogeneous endothelium covered by multi-layered muscular media, whereas the acellular grafts exhibited a patchy endothelial cell layer and a thinner muscular layer.

**Results:**

show the feasibility and efficacy of pulmonary artery reconstruction using clinically available grafts engineered with allogeneic VSMCs in growing swine.

## 1 Translational perspective

Nowadays, many CHD are diagnosed prenatally thus offering the possibility of predesigning an interventional strategy for definitive correction. In this study, we used autologous neonatal blood-derived cells to generate a living conduit and reconstruct the pulmonary artery in piglets. The cellular graft surpassed the acellular control in both primary efficacy endpoints, showing durable and incremental corrective potential and thus opening new avenues for the definitive treatment of pulmonary artery defects. We envision this approach could immediately be implemented in current surgical standards and, in the future, be combined with similar bio-prosthetic solutions for leaflet replacement and RVOT reconstruction.

## 2 Introduction

Congenital heart disease (CHD) is the most common and life-endangering congenital disability worldwide, with a reported number of 1.35 million (M) new cases each year, of which ~5000 in the UK alone. Graft failure is the cause of a sizable subset of re-intervention with remarkable impact on a patient's quality of life.

The ideal cure for patients with Tetralogy of Fallot (ToF) or pulmonary artery atresia, consists of surgical reconstruction of right ventricle outflow tract (RVOT) and implantation of a prosthetic pulmonary conduit. [1] Xenografts, made with bovine or swine valvular, pericardial or intestinal material, represent the most common biological prostheses. Their manufacture includes chemical treatments to remove resident immunogenic cells and strengthen mechanical properties. [[2], [3], [4]] Nonetheless, incomplete decellularization might lead to adverse host immune reaction, while aggressive treatment could remove extracellular matrix (ECM) component crucial for *in vivo* recellularization [5,6].

Despite improving mechanical resistance, fixative treatment of the decellularized tissue during graft manufacturing resulted in poor compliance and calcification after *in vivo* implantation. [7,8] These limitations make the graft unable to grow at the same pace as an infant's heart, resulting in anatomical and functional deterioration within a few years. [9,10]

Tissue engineering holds promises to surpass the current limits of decellularized grafts. [11,12] Successful preclinical studies using prosthetic valve leaflets and swine intestinal submucosa conduits seeded with autologous endothelial cells (ECs)/endothelial progenitor cells (EPCs) [13,14] have been followed by first-in-human studies that confirmed feasibility and advantages of endothelialized grafts. [15,16] Despite the observed improvements of this conduit endothelialisation strategy, the physiological characteristics remained suboptimal in comparison to native vessels, due to the lack of vascular smooth muscle cells (VSMCs). VSMCs are crucial in vessel structure and function; nonetheless, few studies have tested the *in vivo* potential of tissue-engineered vascular grafts (TEVG) repopulated with VSMCs. [17,18]

Despite noticeable progress, including the use of bioreactors to allow the full maturation of TEVG, [19,20] tissue engineering technology still poses major challenges in neonates and infants. The ideal cell population for complex defects should be readily available at birth or require minimal expansion. Mesenchymal stromal cells (MSCs) from the umbilical cord blood (UCB) or neonatal peripheral blood (PB) may have those requisites. [[21], [22], [23], [24], [25]] In addition, the majority of TEVGs tested so far were 10–16 mm in inner diameter, [[26], [27], [28]] i.e. they were oversized for a new-born, considering that, at birth, the pulmonary artery measures ~5–6 mm or even less in pre-term neonates. [29]

Here, we report the results of a randomized, controlled study in piglets, which had the left pulmonary artery reconstructed with small-size conduits engineered with allogeneic VSMCs. At the final 6-month follow-up, the engineered conduits surpassed the acellular conduits on all the considered endpoints, showing proper blood flow velocity, growth potential, and physiological remodelling.

## 3 Methods

### 3.1 Ethical permissions

Twenty human UCB samples were collected at St. Michael Hospital (Bristol, UK), under informed consent from the mother, in accordance with the licence approved by the Southwest Research Ethics Committee (11/HO107/4). The investigation conformed to the principals outlined in the Declaration of Helsinki.

PB was collected within 24 h after birth from Landrace female piglets (N = 18, average weight 1.5 kg) humanely sacrificed using schedule one, following the guidelines of the UK Home Office. Three to four-week-old female Landrace pigs (N = 11, 10–15 kg in body weight) were employed in the *in vivo* graft implantation studies under the UK Home Office ethical approval PPL 30/3019. Animals were treated in accordance with the “Guide for the Care and Use of Laboratory Animals” published by the National Institutes of Health in 1996 and conforming to the “Animals (Scientific Procedures) Act” published in 1986.

### 3.2 Isolation and expansion of MSCs

Blood samples were processed within 5 h from collection. Mononuclear cells (MNCs) were isolated from total human UCB or swine PB using a gradient centrifugation method, previously described by Ingram et al.. [30] Briefly, blood samples were diluted in a double volume of PBS, without calcium and magnesium chloride (Sigma) and loaded on Ficoll-Istopaque columns (GE healthcare). After density gradient centrifugation at 400*g* for 30 min at room temperature, MNCs were removed from the interphase, washed twice in PBS and then treated with 1X Red Blood Cell Lysis Buffer (RBC lysis buffer, Biolegend, diluted with sterile deionized water) for 10 min. Cells were then seeded on culture dishes at a density of 1 × 10^6^/cm^2^ in mesenchymal culture medium, composed by 40% (v/v) LG-DMEM and 40% (v/v) MCDB 201 (both from Sigma), supplemented with 15% FBS (Hyclone), 1% (v/v) penicillin-streptomycin, 1% (v/v) insulin-transferrin-selenium (ITS, Invitrogen), 0.1 mM L-ascorbic acid-2-phosphate, 50 nM dexamethasone, 1% (v/v) linoleic acid–BSA (all from Sigma), 10 ng/ml human Platelet Derived Growth Factor-BB (PDGF-BB, R&D Systems) and 10 ng/ml human Epidermal Growth Factor (EGF, R&D Systems). The resulting cell culture was kept at 37 °C in a humidified 5% CO_2_ incubator. After three days, non-adherent cells were removed by changing the culture medium. The medium was changed twice a week until colonies were visible. Adherent cells were left to grow up to 80% confluence before passaging.

### 3.3 Differentiation of MSCs into VSMCs

MSCs were cultured for 4–12 days in a medium containing 60% (v/v) LG-DMEM (Sigma) and 40% (v/v) MCDB 201 (sigma), supplemented with 1% FBS (Hyclone), 1% (v/v) penicillin-streptomycin，; 1% (v/v) insulin-transferrin-selenium (ITS, Invitrogen), 0.1 mM L-ascorbic acid-2-phosphate (Sigma-Aldrich), 50 nM dexamethasone (Sigma), 1% (v/v) linoleic acid–BSA (Sigma) and 5 ng/ml Transforming Growth Factor-β1 (TGF-β1, R&D). At the end of the induction, the expression of VSMC-specific proteins, including Smooth Muscle Myosin, Heavy Chain 11 (SM-MHC), Alpha Smooth Muscle Actin (aSMA), and Calponin 1 (CNN1), was evaluated by immunocytochemistry (Table 1).

### 3.4 Graft cellularization and maturation in a bioreactor

Decellularized porcine small intestinal submucosa (CorMatrix^®^ Cardiovascular, Inc, USA) was seeded with MSCs at a density of 5 × 10^5^/cm^2^ and maintained under static conditions for 5 days. The engineered-graft was then stitched to the rotating arm of an InBreath bioreactor (Harvard Apparatus, USA) as to fashion a conduit-shape with the cells facing the outer side of the graft. The rotation was set at 1.5 rpm for the first 24 h, and then at 2.5 rpm for 9 days.

### 3.5 In vivo studies

A total of 11 piglets entered a randomization protocol for implantation of conduit-shaped grafts in the left pulmonary artery (N = 6 unseeded CorMatrix; N = 5 allogeneic VSMC-seeded-CorMatrix). The cellularized grafts were engineered with swine allogeneic cells collected from 5 different donors. Surgical procedures were performed with swine under general anesthesia (Ketamine/Midazolam/Dexmedetomidine, Isoflurane) and neuromuscular blockade (Pancuronium Bromide). Details of the operations are reported in the Supplementary Video. Briefly, a left posterolateral thoracotomy was performed; the proximal and distal part of the left pulmonary artery (LPA) (just before the upper and middle lobe branches of the LPA) was clamped and a 3–4 mm of the LPA was resected to accommodate the conduit-shaped graft (~10 mm long and ~6 mm diameter). Animals were allowed to recover under intense postoperative monitoring for the initial 24 h. Analgesic (Paracetamol, Morphine) and antibiotics (Cefuroxime) were administered during this period according to the needs.

Supplementary video related to this article can be found at https://doi.org/10.1016/j.biomaterials.2019.119284.

The following is the supplementary data related to this article:

Video 1 Video 1

Imaging studies were performed using a two-dimensional Doppler Echocardiography system (VividQ, GE Healthcare) prior the graft implantation and at 3 and 6 months thereafter to assess the graft patency and pulmonary blood flow velocity (primary endpoints). After 6 months of follow-up, swine were euthanized by an overdose of IV pentobarbitone according to the surgical facility standard protocols. The main pulmonary artery and its left (where the graft was implanted) and right branches (serving as an internal control) were dissected from the heart and then fixed in 4% PFA or fresh-frozen in liquid nitrogen.

### 3.6 Post-mortem analyses

Tissue samples were washed in PBS, fixed in 4% PFA, moved into cassettes (Histosette I, Simport) processed in a Shandon Excelsior (Thermo), and embedded in paraffin using a Shandon Histocentre 3 (Thermo). The resulting blocks were sectioned at 5 μm for histology and immunohistochemistry (IHC) assessments. For studies performed on cryosections, tissues were washed in PBS, frozen and sectioned at 8 μm in a cryostat (Leica). Hematoxylin and eosin (H&E), Russel-Movat pentachrome and Van Gieson's (EVG) stainings were performed using a Varistain apparatus (Thermo). For immunocytochemistry, paraffin-embedded sections were deparaffinized by clearene washing and rehydrated through an alcohol gradient. A heated antigen retrieval was used for antigen unmasking with 0.01 M citrate buffer, pH 6.0 heated to boiling. Sections were blocked with goat serum and stained with non- conjugated primary antibodies (Table 1) and conjugated- isolectin (Life Technologies) overnight. Cy2 and Cy3 labeled secondary antibodies (Dako) were employed to detect primary antibodies. Slides were mounted with DAPI Hardset mounting medium (Vectashield). Images were taken with a Leica SP5 confocal microscope using a 20x or 40x oil immersion objective.

**Table 1:** Antibodies used in Immunocytochemistry.

Antibody	Company	Dilution	Catalog #
Anti-Actin, α-Smooth Muscle (α-SMA)	SIGMA	1:100	A5228
Calponin (CNN1)	Dako	1:100	M3556
Smooth Muscle Myosin Heavy Chain (SM-MHC)	R&D Systems	1:100	IR066

For electron microscopy analyses, PFA-fixed samples were washed in 0.1 M phosphate buffer and then fixed in 25% osmium tetroxide in phosphate buffer. After washing in phosphate buffer, samples were dehydrated through an alcohol gradient ending with three changes of absolute alcohol. Samples were dried in a critical point dryer (Leica EM CPD300). They were finally coated at 100 mA for 30 s using EMITECH K575X sputter coater and observed using a Quanta 400 FEI scanning electron microscope.

For smooth Muscle Actin quantification, the graft regions composed of smooth muscle cells were identified immunohistochemically by quantification of Smooth Muscle Actin expression in 10 random fields per section under a 20X magnification lens. ImageJ software was used to quantify the percentage of positive cells in the remodelling acellular and cellular grafts. The ImageJ function threshold was applied in the channel of interest to convert each image to a binary version, and pixels representing smooth muscle regions were quantified, pooled together and converted into mm [2]. The smooth muscle area was then divided by the total area of the newly formed tissue. Moreover, the Smooth Muscle Actin-stained samples were employed to measure the thickness of the smooth muscle cells layer that developed in the acellular and cellular graft tissue after 6 months *in vivo*.

### 3.7 Statistics

Average values are plotted with ‘n’ value shown in figure legends. Animal demographics and echocardiographic data are expressed as mean ± SD. Statistical significance for differences between experimental groups was determined using Student's *t*-test when comparing two groups and ANOVA with *post-hoc* when comparing more than two groups. Asterisk symbols are used in figures to represent the statistical difference between groups. Any reference to a difference between groups implies statistical significance, at least, at the level of *P* < 0.05.

## 4 Results

### 4.1 Maturation of the cellularized TEVG in a dynamic bioreactor

**Fig. 1:**
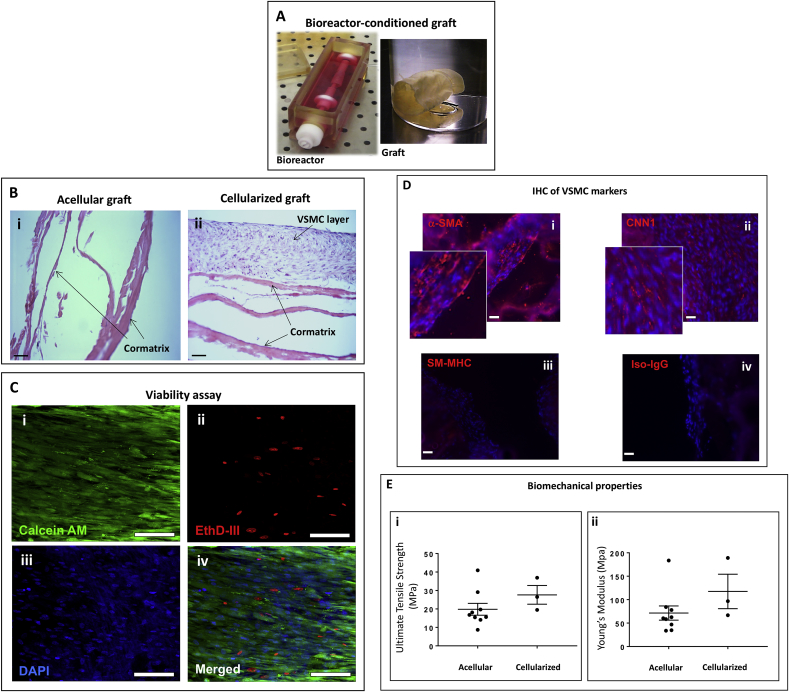
Maturation of human VSMC-engineered grafts in a dynamic bioreactor. (A) Photograph of the bioreactor and the produced cylindrical scaffold. (B) H&E staining of acellular (i) and cellularized (ii) grafts after conditioning in the bioreactor (Bar = 50 μm). (C) Fluorescence microscopy images of viable cells (Calcein AM staining, green, n = 3) and dead cells (EthD staining, red). Nuclei are stained blue by DAPI. (bar = 50 μm). (D) Representative fluorescence microscopy images of VSMC markers. (bar = 50 μm). (E) Ultimate tensile strength (i) and Young's Modulus (ii) of acellular and cellularized grafts. Cells from three donors (n = 3) run in triplicates were tested in this study.

**Fig. 2:**
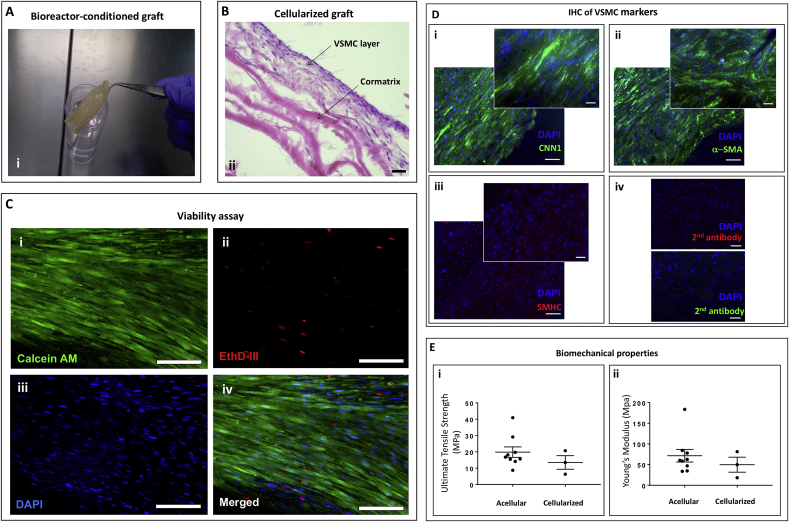
Swine VSMC-engineered grafts maturation in a dynamic bioreactor. (**A**) Photograph of the cylindrical scaffold from the bioreactor. (**B**) H&E staining of cellularized grafts after conditioning in the bioreactor (bar = 50 μm). (**C**) Representative fluorescence microscopy images of viable cells (**i**, Calcein AM staining, green, n = 3) and dead cells (**ii**, EthD staining, red). Nuclei are stained blue by DAPI (**iii**). Merged staining (**iv**). (bar = 50 μm). (**D**) Representative fluorescence microscopy images of VSMC markers CNN1 (**i**), a-SMA (**ii**) and SMHC (**iii**). Controls for the secondary antibodies (**iv**). (bar = 50 μm). (**E**) Ultimate tensile strength (**i**) and Young's Modulus (**ii**) of acellular and cellularized grafts. Cells from three donors (n = 3) run in triplicates were tested in this study.

Having confirmed the ability of MSCs to differentiate into VSMCs *in vitro* (Data in Brief article), we next attempted to achieve the same result with human or swine MSCs seeded in a conduit graft and maintained in a bioreactor to prime the cells to the dynamic conditions they would be exposed to *in vivo*. Such an integrated approach allows a more rapid maturation of the TEVG and reduces the risk of contamination associated with repeated cell passaging. The protocol was carried out in two steps. First, we seeded human or swine MSCs onto a flat Cormatrix sheet and maintained the cellularized graft in a mesenchymal culture medium under static conditions for 5 days. At the end of this period, we shaped the graft into a cylindrical conduit with the cells facing the outer side of the graft and then stitched it to the rotating arm of a dynamic bioreactor containing the inductive media. After 10 days, the conduit was removed from the incubator, photographed (Fig. 1A), and processed for analysis of cell viability and immunohistochemistry. Acellular grafts were run in parallel throughout all the staged process described above. The control graft was confirmed to be acellular (Fig. 1Bi), while the human UCB-MSC-engineered graft contained a multi-layer of cells within its internal structure as assessed by H&E staining (Fig. 1Bii). Imaging of the grafts using markers for live/dead cells demonstrated the viability of the engineered construct (Fig. 1C). Immunohistochemistry studies verified the engrafted cells expressed α-SMA and Calponin but were low abundant in SM-MHC (Fig. 1D). The ultimate tensile strength and Young's Modulus of elasticity were relatively similar in the acellular and cellularized grafts (Fig. 1E). Similar results were obtained in grafts engineered with swine PB-MSCs (Fig. 2A–E). The ultimate tensile strength and Young's Modulus of elasticity (0.46 ± 0.17 and 1.39 ± 0.77 MPa respectively; n = 5) of the native left pulmonary artery (LPA) were over 29 times lower than those of acellular and cellularized grafts.

### 4.2 In vivo studies

**Fig. 3:**
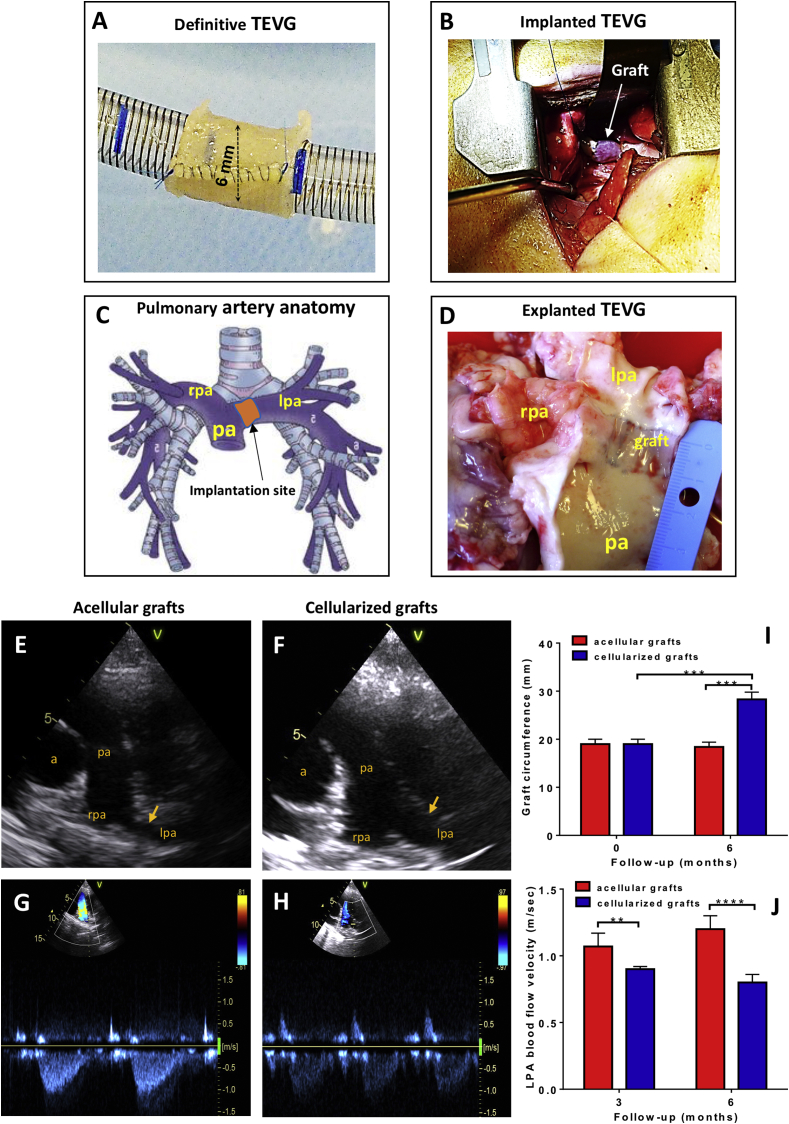
Outcome of the preclinical trial. (**A&B**) The cell engineered graft was shaped into a 6 mm diameter conduit with 10 mm length (**A**) and implanted into swine left pulmonary artery (**B**). (**C**) Anatomy of the main pulmonary artery and its major branches. Arrow indicates the position of TEVG implantation. (**D**) Representative image of the explanted TEVG with main pulmonary artery (pa) and left pulmonary artery (lpa) cut-open to show the luminal side. The graft is marked by the blue anastomosis sutures (rpa, right pulmonary artery). Representative ultrasound images of the pulmonary arteries of acellular and cellularized grafts. Arrows indicate level of graft insertion (a, aorta; pa, pulmonary artery; rpa, right pulmonary artery; lpa, left pulmonary aretry). (**G&H**) Representative images of Colour Doppler blood velocities in acellular and cellularized groups. (**I**) Circumference of the cellularised and acellular grafts at implantation and six months post-surgery. ANOVA with *post-hoc* testing were used. ***p < 0.001. (**J**) Blood flow velocities through the acellular and cellularized LPA at 3 and 6 months of follow-up. ANOVA with *post-hoc* testing were used. **p < 0.01. ***p < 0.001.

Swine cell-engineered and acellular grafts, shaped as 6-mm inner diameter conduits (Fig. 3A) were implanted in the left pulmonary artery of piglets (Fig. 3B–D), according to a randomized, controlled study design. Following surgery, swine grew at a normal rate, and there was no significant difference in body weight gain between animals implanted with cellularized or acellular grafts (124 ± 8.7 kg and 138 ± 2.6 kg respectively) at 6 months post-surgery. Echocardiography showed grafts were patent in both groups (Fig. 3F&G). However, the cellularized conduits showed a lower blood flow velocity as compared with the acellular conduits (Fig. 3H, I&J). The group difference in this primary hemodynamic endpoint was incremental from 3 to 6 months post-implantation (0.17 ± 0.04 and 0.40 ± 0.05 m/sec, P < 0.01 and P < 0.0001, respectively) (Fig. 3K & Table 2). In line with this, at the 6 months assessment, the cellularized grafts had a larger circumference (28.32 ± 2.31 mm) as compared to acellular graft controls (18.37 ± 2.10 mm, P < 0.001) (Fig. 3K). Moreover, the circumference of the tissue-engineered pulmonary artery was 50% larger than the graft before implantation (P < 0.05), whereas no dimensional change was observed regarding the acellular graft (P=N.S.) (Fig. 3G). Calculation of the left/right pulmonary artery inner diameter ratio, which uses the untouched side as an internal control for physiologic remodelling, showed higher values in animals implanted with cellularized conduits (0.74 ± 0.07) as compared with acellular conduit controls (0.48 ± 0.04, P < 0.0001). Altogether, these results indicate that cellularization confers growing capacity to the TEVG thereby significantly reducing the mismatch with the contralateral artery. In both groups, we observed no ventricular hypertrophy or tricuspid regurgitation (data not shown).

**Table 2:** Pulmonary arteries size and blood velocity in animals at 3 and 6 months.

	Acellular 3 months	Cellularized 3 months	P value	Acellular 6 months	Cellularized 6 months	P value
LPA graft diameter (mm)	5.02 ± 0.4	7.5 ± 0.6	0.0003	6.03 ± 0.7	9.1 ± 0.6	0.0001
LPA graft velocity (m/sec)	1.07 ± 0.1	0.9 ± 0.02	0.06	1.2 ± 0.1	0.8 ± 0.06	0.0006
RPA diameter (mm)	10.6 ± 0.6	11.7 ± 0.7	0.09	12.6 ± 2.2	12.3 ± 0.7	0.8
Main PA diameter (mm)	13.9 ± 1.5	15.4 ± 1.2	0.2	16.0 ± 1.6	16.6 ± 1.6	0.5

Values are Means ± SD.

**Fig. 4:**
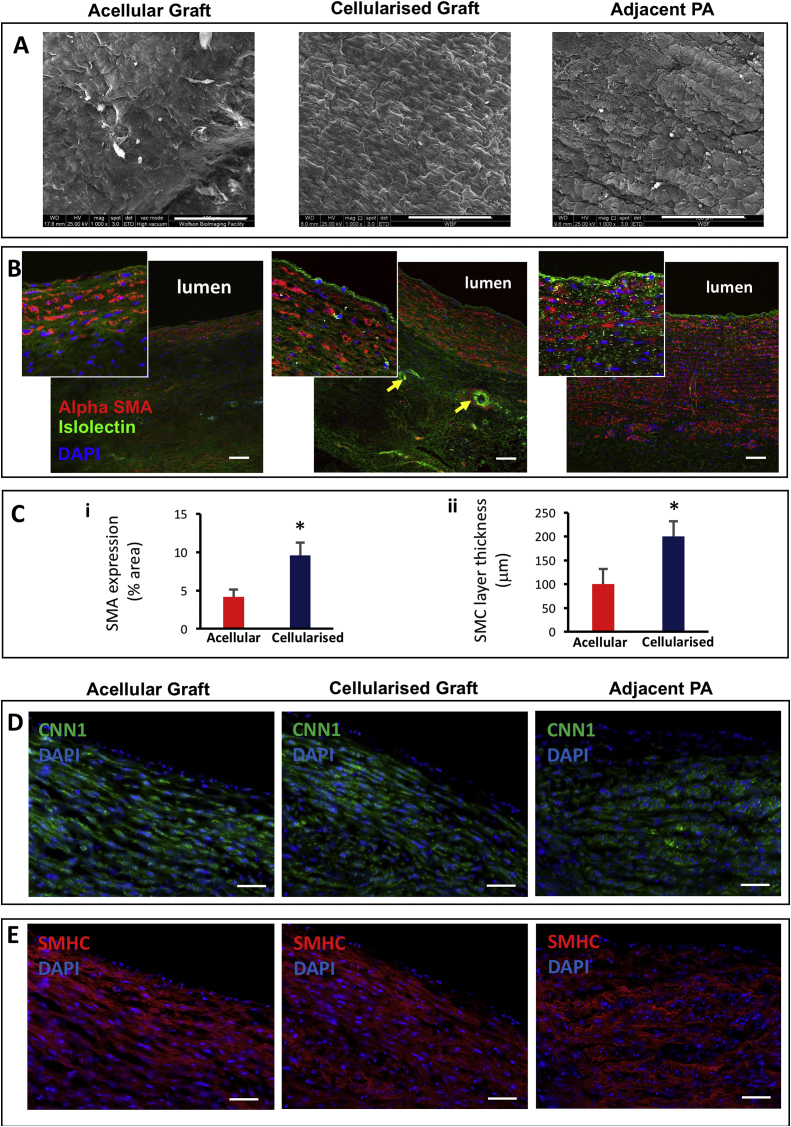
Electron scanning microscopy and immunohistochemistry of graft explants. (A) Electron microscopy images showing the internal surface of the explanted acellular graft compared with the cellularized graft and the pulmonary artery adjacent to the graft (Scale bar = 100 μm). (B) Fluorescence microscopy images showing endothelial cells (isolectin, green) lining the internal lumen and surrounding layer of VSMCs (αSMA, red). Arrows indicate vessels (Scale bar = 90 μm). (C) SMA expression quantification and smooth muscle cells (SMC) layer thickness in acellular and cellularised grafts. Student's t-test was used. *p < 0.05. (D) Representative images of the immunofluorescent staining of CNN1 in explanted acellular and cellularized grafts (Scale bar = 50 μm). (E) Representative images of the immunofluorescent staining of SMHC in explanted acellular and cellularized grafts (Scale bar = 50 μm). The adjacent left pulmonary artery was used as a control sample.

**Fig. 5:**
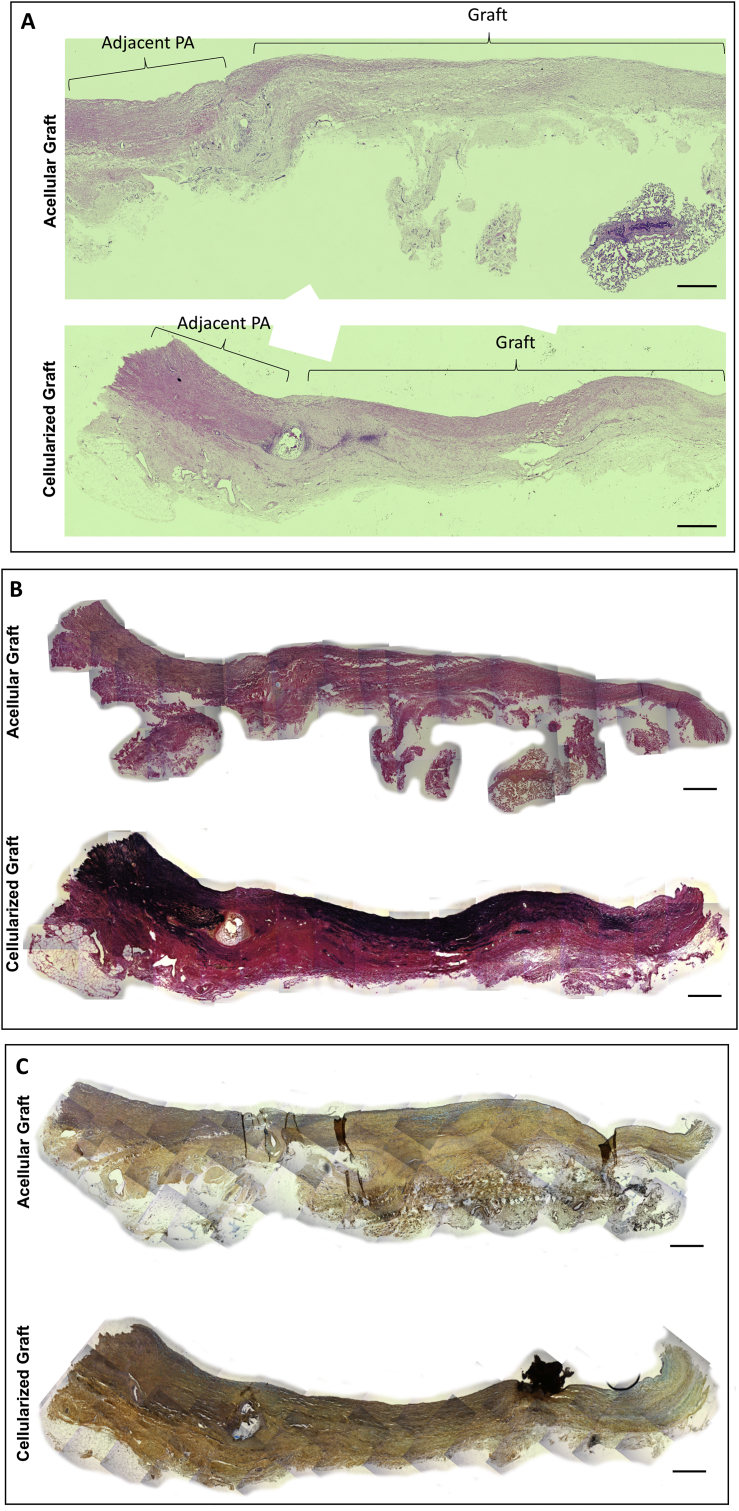
Histology of graft explants. Representative histological images of H&E (A), EVG (B) and Russel-Movat pentachrome (C) stainings in explanted acellular and cellularized grafts (Scale bar = 500 μm).

Electron microscopy of the luminal graft side showed a well-organized endothelial layer in the cellularized grafts that was similar to the endothelium of the neighbouring pulmonary artery (Fig. 4A). In contrast, the luminal side of the acellular graft looked patchy suggesting an incomplete endothelialization (Fig. 4A). The IHC studies of explanted grafts using endothelial and smooth muscle markers confirmed the cellular grafts had a clear endothelial cell layer similar to the neighbouring pulmonary artery, and a significantly thicker layer of VSMCs as compared to the acellular graft (Fig. 4B–E). The cellularized grafts also showed a significantly higher percentage of SMA-stained cells than acellular grafts (Fig. 4C). Additionally, the seeded grafts exhibited isolectin-positive microvessels surrounded by VSMCs in the outer layer, resembling the formation of adventitial *vasa vasorum* (Fig. 4B). Furthermore, the histological analysis demonstrated that the CorMatrix was no longer visible after 6 months, suggesting complete absorption and integration of both the acellular and cellularized graft into the host surrounding tissue (Fig. 5 A-C). Besides, the EVG and Russel-Movat pentachrome staining showed extracellular matrix proteins (collagen, elastin, GAGs and proteoglycans) released by the newly formed tissue in both the acellular and cellularized grafts (Fig. 5 B-C). In support of the previous quantification results of Fig. 4C, the cellularized grafts presented a greater nuclei infiltration in comparison to the acellular constructs (Fig. 5). In addition, elastin staining looked higher in cellularized grafts (Fig. 5 B).

**Fig. 6:**
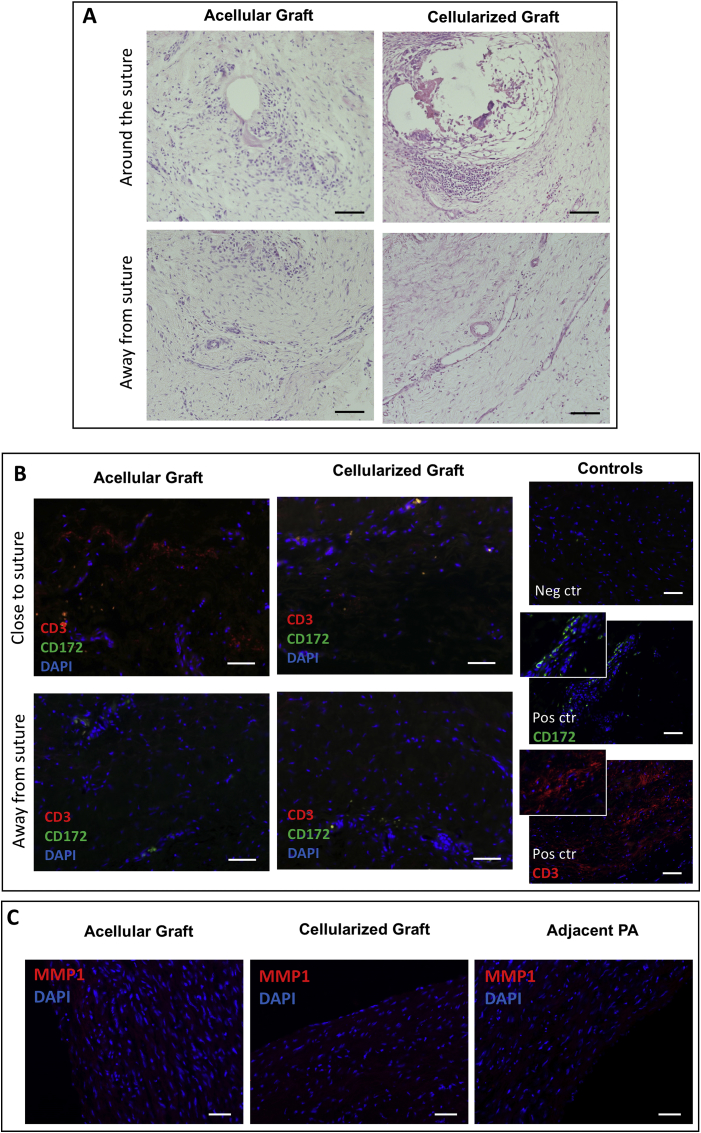
**(A)** Representative images of the histology in explanted acellular and cellularized grafts around suture area and away from it (Scale bar = 50 μm). (**B**) Representative images of the immunofluorescence staining of T-cell marker CD3 and granulocyte & macrophage marker CD172 in explanted acellular and cellularized grafts (Scale bar = 50 μm). Negative (Neg) and positive (Pos) controls (ctr) are shown. Lymph node was used as positive control. Nuclei are counterstained by DAPI. (**C**) Representative images of immunostaining of remodelling marker MMP1 in explanted acellular and cellularized grafts (Scale bar = 50 μm).

No difference in the inflammatory cell infiltration was observed in the acellular and cellularized grafts (Fig. 6A and B). Inflammation was especially observed around the sutures mainly with the presence of lymphocytes, granulocytes and macrophages (Fig. 6A and B). This inflammatory response was milder away from the suture and throughout the grafts with few infiltrating cells, mainly lymphocytes (Fig. 6A and B). Immunostaining of the remodelling marker, MMP1, showed low expression levels in the acellular and cellularized grafts (Fig. 6C). Evaluation of markers for osteogenic (Alizarin Red), adipogenic (Oil Red O) and chondrogenic (Alcian Blue) demonstrated similar expression in explanted acellular grafts, cellularized grafts, and the LPA suggesting that the implanted cells did not differentiate into these three lineages (Data in Brief article).

## 5 Discussion

This study demonstrates for the first time the growth capacity of a TEVG functionalized with MSC-derived VSMCs after implantation into the left pulmonary artery of piglets. We used a porcine model because its cardiovascular system has a similar anatomical structure and physiological characters to humans. Another fundamental feature that makes swine popular in regenerative medicine is their rapid growing speed. Considering a Landrace piglet grows into an adult size pig in 6 months, we decided this would be an adequate follow-up duration to compare cellularized and acellular Cormatrix conduits in an efficacy study.

Cormatrix consists of decellularized porcine small intestinal submucosa that has been processed to eliminate all cells while retaining the structural extracellular matrix proteins. Clinical use of Cormatrix has been introduced since 2006 for pericardial closure and cardiac tissue repair with encouraging early results. [31] However, recent evidence suggest a high rate of intimal hyperplasia formation and stenosis with Cormatrix implantation in the low-pressure small-diameter vasculature. [32] Our *in vitro* study indicates that Cormatrix has supportive properties and biocompatibility with both human and swine MSCs. *In vivo*, the acellular conduit failed to match the contralateral pulmonary artery, but we could not see neointima formation. We also found that the endothelialization was incomplete, with only patchy and non-organized luminal distribution. The cellularized graft outperformed the acellular graft on all predefined efficacy endpoints. In particular, the cellular grafts grew in keeping with the animal growth, showing a lower mismatch with the contralateral untouched pulmonary artery as compared with the acellular graft, and proper endothelialization.

VSMCs are mature somatic cells with limited *in vitro* proliferation ability. They are not only the major cell component of a blood vessel but also play key roles in maintaining blood vessel function, morphology and restoration. Most of the progress in functional VSMC production is achieved by using bone marrow-derived MSCs or pluripotent stem cells. However, these competitive solutions are less attractive than expanding MSCs from the PB as we did in the present study. Indeed, bone marrow harvesting requires an invasive procedure, while immaturity of pluripotent-derived cells may carry the risk of cancerous transformation. Autologous/allogeneic MSCs from new-borns and infants have qualitative and quantitative properties that make them optimal candidates for regenerative medicine, especially if the recipient is also a young individual. We demonstrated that these juvenile characteristics confer PB MSCs with a rapid growth rate enabling rapid expansion and graft cellularization given the urgent correction of severe cardiac defects.

Our unique approach for perinatal MSC maturation into VSMCs during the graft incubation in a bioreactor offers scope for the prompt functionalization with specialized cells and also allows the control of undesired MSC differentiation into calcific or adipogenic elements that could trigger an *in vivo* adverse remodelling. Flow cytometry analysis identified the expression of mesenchymal markers in the source MSC population while confirming the absence of endothelial and hematopoietic markers, which reassures on the purity of the cell product.

Results of our *in vivo* study have important clinical implications for the treatment of complex congenital cardiac disease such as pulmonary atresia with hypoplastic native pulmonary arteries and major aorto-pulmonary collaterals. Surgical rehabilitation of the pulmonary arteries is the only option in this clinical scenario using different type of prosthetic materials. This has been associated with a high incidence of conduit-related complications, particularly with smaller conduits, such as neointimal proliferation, thrombosis, calcification, chronic inflammation and lack of growth. For this reason, although surgical interventions have been employed successfully for a number of years, the long-term prognosis has remained poor with an increased incidence of complications, repeated open-heart surgical procedures and sudden death. [33] Our study is the first to demonstrate the efficacy of a decellularized scaffold repopulated with perinatally-harvested cells to reconstruct a small pulmonary artery (6 mm in diameter) and its capacity to grow and remodel in an *in vivo* large animal model. Similar promising results were recently reported by Syedain et al. who demonstrated somatic growth and normal physiological function for nearly 1 year of a TEVG implanted in a growing lamb model. [28] The main limitation of their model was that they did not test the small pulmonary arteries but the main pulmonary artery (16 mm in diameter), the former and not the latter representing the “Achilles heel” of congenital conditions, such as pulmonary atresia with hypoplastic pulmonary arteries. Indeed, large diameter vessel substitutes made of synthetic or biological tissue have been successfully employed by surgeons, whereas, such synthetic prostheses generally fail as a substitutes of medium to small diameter (<6 mm) vessels. [34] Furthermore, Syedain et al. had to use subdermal heparin for the duration of their study in order to reduce the risk of clotting while no such therapy was required in our small TEVG. [28]

Tunica media is the thickest layer of large blood vessel wall (like pulmonary artery), mainly composed of VSMCs and elastic fibres, which regulates the blood flow and pressure as well as provides most mechanical strength. Regeneration of tunica media is the key part of large diameter blood vessel tissue engineering. Therefore, the primary successful endpoint of this study was to construct a live functional tunica media *in vitro*, which persisted after transplantation of the TEVG *in vivo.* This could be the combined result of the initial *in vitro* cellularization followed by proliferation of donor and recipient VSMCs after implantation. In contrast, the recruitment of endogenous myogenic cells was the only operative mechanism allowing the formation of a thin muscular layer within the unseeded graft wall.

We also observed intriguing phenomena with regard to luminal endothelialization and formation of muscularized capillaries in the external layer of the cellularized graft. These effects are likely due to paracrine stimulation of endothelial cell recruitment from both the intima layer and the adventitial *vasa vasorum* of the grafted pulmonary artery. A conversion of VSMCs into endothelial cells through mesenchymal endothelial transition cannot be excluded, but this possibility warrants further investigation.

Immune response is a major challenge for a successful integration of a TEVG. We observed some inflammatory cells mainly located near the sutured area, which suggests the TEVG did not trigger a major immune response by the host. Remarkably, the graft maintained structural integrity at 6 months post-surgery, which is in line with the observed low expression of the remodelling marker.

## 6 Conclusions and study limitations

The molecular mechanism underlying the observed advantage of using cellularized-engineered grafts remains to be investigated. Although the duration of the study covered the complete maturation of piglets into adult animals, we cannot predict the grafts would remain functional over the entire life, which is the ultimate clinical outcome. In addition, one major limitation of our study is that the implanted graft was placed under normal PA pressure. While that will be the case for some patients, more severe conditions characterized by high vascular resistances and pulmonary hypertension may affect the cellularized graft durability. Further research is needed to confirm the viability of our approach under such complex situations. Another limitation of our study is that we could not assess the tensile properties of the graft after 6 months from the implantation because such an assessment would have precluded the histological analysis. However, the integrity of the explanted grafts reassured us the graft was strong enough to withstand physiological blood pressure levels. Furthermore, the pulmonary artery is just one of the anatomical components to be corrected in complex CHD. Translation of our approach to the leaflets and RVOT would require additional implementations, in particular, the use of other specialized cells. We envision that TEVG-based reconstruction of the pulmonary artery should be tested alone in a first-in-human clinical trial to maximize the benefit and minimize the risk of the new procedure and implemented later with similar bioprosthetic solutions.

## Author contributions

MC, AH, MTG conceived and designed the research. MTG, HJ, MMS, DI, AA, CZ, DHD performed experiments. MTG, HJ, MMS, DI, AA analysed data. MC, MTG, HJ, MMS, DI, AA, and PM interpreted results of experiments. MTG, HJ, MMS, DI, AA, PM prepared figures. MC, MTG, HJ, MMS, DI, AA, AH and PM prepared the manuscript. All authors read and approved the final manuscript.

## Conflicts of interest

The authors have no conflicts of interest to disclose.

## Funding

This study was supported by grants from the Sir Jules Thorn Charitable Trust (MC, PM), the Enid Linder Foundation (MC, MTG, AH), the British Heart Foundation (MC), the NIHR Bristol Biomedical Research Centre in Cardiovascular Medicine (MC), and the Medical Research Council (PM, MC, MTG).
